# Study of AEB and active seat belt on driver injury in vehicle–vehicle frontal oblique crash

**DOI:** 10.1038/s41598-023-48729-7

**Published:** 2023-12-18

**Authors:** Pingfei Li, Yi Lei, Jingqian Liao, Daowen Zhang, Xinchi Dong, Tianshu Zhang

**Affiliations:** 1 School of Automobile and Transportation, Xihua University, Chengdu, 610039 China; 2 Vehicle Measurement Control and Safety Key Laboratory of Sichuan Province, Xihua University, Chengdu, 610039 China; 3 Engineering, Computer and Mathematical Sciences, The University of Adelaide, Adelaide, 5005 Australia

**Keywords:** Mechanical engineering, Computational science

## Abstract

The safety of vehicle occupants in oblique collision scenarios continues to pose challenges, even with the implementation of Automatic Emergency Braking (AEB) systems. While AEB reduces collision risks, studies indicate it may heighten injury risks for out-of-position (OOP) occupants. To counteract this issue, the integration of active seat belts in vehicles equipped with AEB systems is recommended. Firstly, this study established an oblique angle collision scenario post-AEB activation using data from the Chinese National Automobile Accident In-depth Investigation System (NAIS) database, analyzed through Prescan software. The dynamic response of the vehicle was examined. Following this, finite element (FE) models were validated to assess the effects of collision overlap rate, AEB braking strategy, and active seat belt pre-tensioning on occupant injuries and kinematics. Under specific collision conditions, the impact of the timing and amount of seat belt pre-tensioning, as well as airbag deployment timing on occupant injuries, was also explored. Findings revealed that a 75% collision overlap rate significantly increases driver injury risk. Active seat belts effectively mitigate injuries caused by OOP statuses during AEB interventions, with the lowest Weighted Injury Criterion (WIC) observed at a pre-tensioning time of 200 ms for active seat belts. The study further suggests that optimal results in reducing occupant injuries are achieved when active pre-tensioning seat belts are complemented by appropriately timed airbag deployment.

## Introduction

Vehicle-to-vehicle frontal collision is a common type of traffic accident. According to the National Highway Traffic Safety Administration (NHTSA), the United States witnessed a total of 13,066 fatal vehicle-to-vehicle accidents in 2019, out of which 6,039 were diagonal collisions^1^. Though AEB has shown effectiveness in reducing crashes by 27% to 38%^2,3^, some studies have indicated that AEB may lead to situations with out-of-position (OOP) occupants. This can compromise the effectiveness of the vehicle's restraint system, consequently elevating the risk of injury to the occupants^4,5^. An increasing number of vehicles have advanced assisted-driving features, such as AEB and automatic emergency steering (AES). In the development of the restraint system, the occupant posture is assumed to be a normal seating position, the posture which is defined in the regulations. However, during driving, the occupant may be in an "OOP" status due to the intervention of advanced assisted driving. Once a collision occurs, the restraint system may not be able to protect the occupant effectively. Hence, the integration of active and passive safety technologies in vehicles plays a crucial role in enhancing vehicular safety. This paper aims to explore the impact of AEB and active seat belts on reducing occupant injuries in scenarios of frontal oblique collisions between vehicles. Furthermore, it delves into the optimization of active seat belt parameters to maximize occupant safety.

To reduce the casualties of occupants in oblique collisions, countries around the world have contributed to regulations and standards on frontal oblique collisions. In the United States, regulations pertaining to diagonal crashes were first introduced in the 1980s through the FMVSS208, with ongoing updates to the testing requirements^6^. The European ECE R94 regulation, established in 1995, mandated a 30° angled frontal crash test at a speed of 50 km/h^7^. Researchers have used various methods to investigate occupant injuries in oblique-angle crashes. Rudd et al.^8^ screened and analyzed over 380 crashes that occurred in the United States and reported that occupants’ pelvis and thorax were the most vulnerable areas in an oblique-angle collision. Zhang et al.^9^ used finite element analysis to show that the severity of head and neck injuries has a positive correlation with the initial velocity in oblique collisions. López-Valdés et al.^10^ used cadaver experiments to study the kinematic response of the human body in oblique-angle collisions and demonstrated that these collisions increased the lateral movement of the human torso; this movement can cause fatal neck and chest injuries. Paul et al.^11^ indicated that chest injuries are a cause of death and serious injury in car accidents, calling for the development of new injury assessment tools. In addition, Barbat et al.^12^ analyzed 31 cases of frontal oblique collision tests and reported that occupant injury in a frontal oblique collision was correlated with vehicle type. Zeng et al.^13^ demonstrate that compared to cars, vans, pickup trucks, light trucks, medium trucks, and heavy trucks have better self-protection and stronger aggressiveness. Kongwat et al.^14^ investigated crashworthiness and safety assessment of L7e vehicles and called for additional crash tests to be added for this type of vehicle.

While the extensive use of AEB has significantly enhanced driving safety, it is not without its drawbacks. Notably, in instances where AEB is activated but fails to prevent a collision, the resulting OOP scenario can exacerbate the driver's injuries^15,16^. To address this issue, some researchers advocate the use of active seatbelt technology. This technology proactively retracts the seatbelt spool, tightening the seatbelt and thereby reducing the forward displacement of the occupant during the pre-collision phase, effectively mitigating the risk of being OOP^17,18^. Radu et al. emphasized that optimal protection is achieved when AEB, seatbelts, and airbags are activated simultaneously^19^. Conventionally, passive and active vehicle safety has been treated as separate domains. However, in the context of evolving vehicle usage patterns and heightened safety standards, the integration of both passive and active safety technologies has become increasingly indispensable.

Integrating active and passive safety technologies involves comprehensively considering the coupling of these two by analyzing the pre-crash phase and post-crash phase as a single process. Hu et al.^20^ evaluated injuries in simulated frontal oblique collisions with both OOP and normal-position occupants; the study served as a reference for the development of reversible restraint systems. Gan et al.^21^ simulated rear-end collisions in which AEB was applied, studied the interaction between the occupant and the restraint system, optimized the AEB algorithm, and proposed an active pre-tensioning seat belt configuration scheme that takes into account both occupant safety and comfort. Xu et al.^22^ analyzed the kinematic response of an occupant before a crash in a vehicle equipped with both AEB and AES. Another study discussed the protection of occupants by automatic emergency collision avoidance systems and pretrigger restraint systems in unavoidable collisions.

In summary, researchers have investigated the characteristics of occupant injuries in oblique-angle collisions and the effects of AEB and seatbelts on the kinetic response of and injury to occupants; they have also simulated passive-active safety system integration to investigate occupants’ kinematic response and injury in various scenarios. However, further research is needed to determine the effect of integrating AEB with active seat belts on occupant injury in vehicle–vehicle frontal oblique collisions. In this work, the AEB scenario and collision finite element model were established. The dynamic responses of the vehicle in the pre-crash phases and post-crash phases were investigated, and the dynamic responses of the two phases were combined as the boundary conditions of the finite element model to predict the damage to the driver caused by different AEB control strategies and collision overlap rates. Based on these results, the effect of active seatbelt parameters on driver injury was assessed by conducting several simulations. The results can facilitate the optimization of the coupling between AEB and restraint systems, and the method can be a reference for active-passive safety technology applied in other crash scenarios.

## Methods

### Date resource

The National Automobile Accident In-Depth Investigation System (NAIS) of China was established in August 2011 by the Defective Products Management Center and several vehicle accident research institutions and motor vehicle forensic identification centers to handle vehicle safety in China, such as automobile recalls, vehicle accident research, and automotive product safety improvement. Currently, the NAIS has developed seven in-country vehicle accident deep survey collection sites. The NAIS database contains detailed information–such as crash participants, damage to vehicles and people, and the road environment–on various types of traffic accidents and hazardous vehicle incidents, including vehicle fires^23^. These crash data enable reconstruction of the crash process and in-depth analysis of the causes of incidents. Because the cases recorded in the database are realistic and representative, the database has been used as an investigative resource by scientists^24–26^.

**Figure 1:**
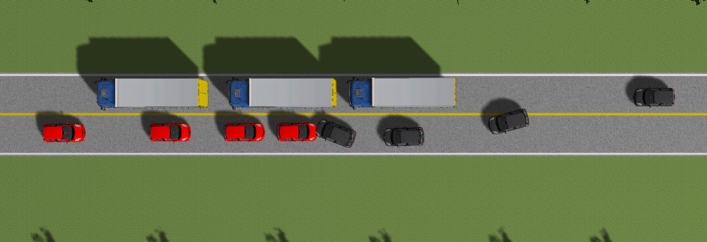
Representative AEB collision.

By using incident keywords in the NAIS database, 740 frontal oblique collisions were identified from 1843 vehicle–vehicle frontal collisions. In these accident cases, death was most commonly caused by severe injury to the head or chest and abdomen. To investigate the mechanism of driver injury in frontal oblique collisions, a representative frontal oblique collision case was selected from these incidents and then simulated. The case was an incident that occurred on a two-way, two-lane road between a main vehicle (Yaris sedan, red) and a target vehicle (black, Fig. 1). In the collision, the target vehicle in the oncoming lane entered the main vehicle’s lane to overtake a large truck; the main vehicle’s AEB detected the target vehicle and was activated. Simultaneously, the driver of the target vehicle attempted to steer to avoid the main vehicle. However, due to the high speed and the short distance between the vehicles, the collision was unavoidable, and a frontal oblique collision occurred. The active security strategy was applied to this case, and PreScan modeling and Simulink were used to control the AEB parameters in the simulation.

The main vehicle in the scenario was modeled using a two-dimensional simple dynamics model, and two technology-independent sensors were used to simulate long-range millimeter-wave radar and short-range millimeter-wave radar. The time-to-collision (TTC) algorithm was employed to control the AEB^27^. In the TTC algorithm, if the radar detects a potential risk ahead on the road, the system provides an alert 2.6 s or less before the predicted crash. If the driver has not intervened 1.6 s before the predicted crash, the system brakes slightly; if the driver has still not attempted to avoid the crash 0.6 s before the predicted crash, the AEB system forces braking to avoid the collision. By simulating this process, the dynamic response of the vehicle in the precrash stage could be obtained.

### FE model

#### Validation of the FE vehicle model

**Figure 2:**
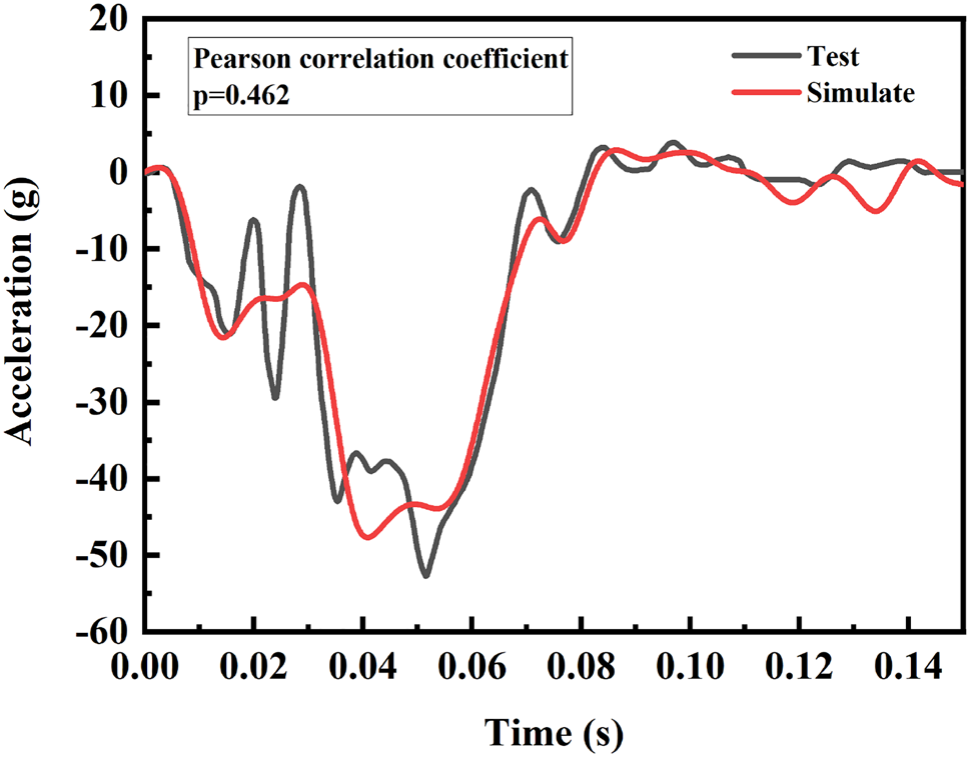
Acceleration of the rear seat cross beam.

A finite element model of the Yaris sedan was developed by the National Collision Analysis Center (NCAC) of George Washington University through reverse engineering. To achieve the goals of this study, an occupant restraint system was added to the model. The frontal collision in the NHTSA New Car Assessment Program (NCAP) report 5677 was simulated and compared with the actual NHTSA NCAP test data from the report. The simulation results were consistent with the physical test results^28^. The time and shape of the simulated and experimental peak acceleration were similar, proving that the modified model is valid (Fig. 2).

#### ATD FE model

The Hybrid III 50th Percentile Male Crash Test Dummy, developed by Livermore Software Technology Corporation in collaboration with the NCAC, was selected for this study. The model comprises over 400,000 parts; the torso sections are connected using hinges to ensure high biological similarity between the dummy and humans. The Hybrid III is the most widely used crash test dummy globally and has been employed by numerous researchers to assess occupant injuries in frontal crash tests. Audrey et al.^29^ used a Hybrid III dummy to simulate the kinematic response of a driver’s upper extremities to investigate damage caused by airbags. Through slide experiments, Devon et al.^30^ demonstrated that the Hybrid III dummy has a favorable kinematic response and biomimetic properties. Kerry et al.^31^ used Hybrid III dummies to assess the effect of various combinations of safety restraint systems on standard sensitivity to chest injury. Peng et al.^32^ used the dummy to investigate injuries to a left-rear passenger in frontal diagonal collisions.

#### Driver-side restraint system FE model

**Figure 3:**
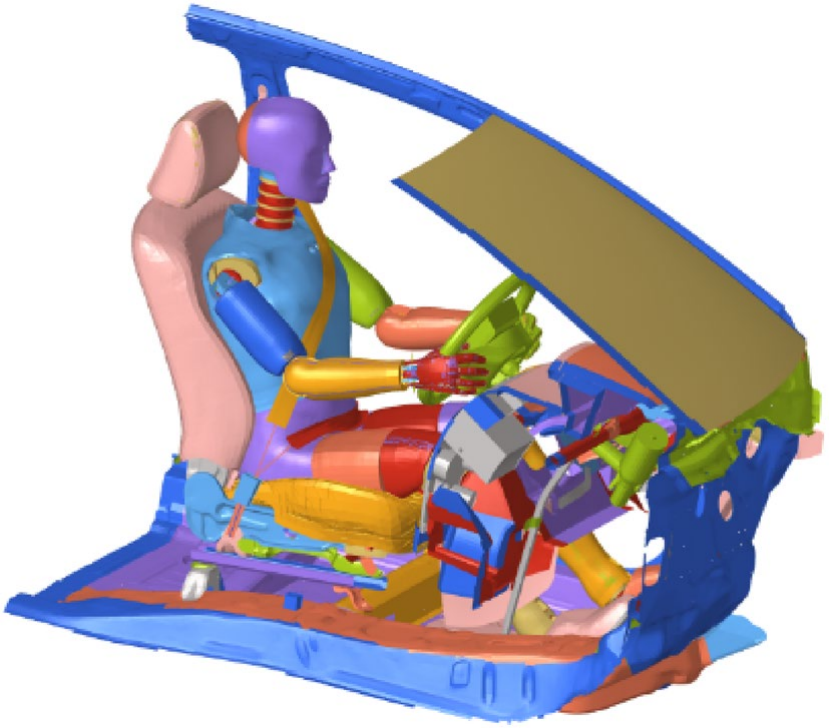
FE model of the driver-side restraint system.

Both the NHTSA NCAP report and the simulation results indicated that the cabin structure remained intact after the collision. Therefore, the model of the driver-side restraint system was split from the Yaris sedan FE model. The entire constrained system model comprised a one-quarter occupant compartment, dummy, seatbelt, airbag, and other relevant vehicle components (Fig. 3).

**Figure 4:**
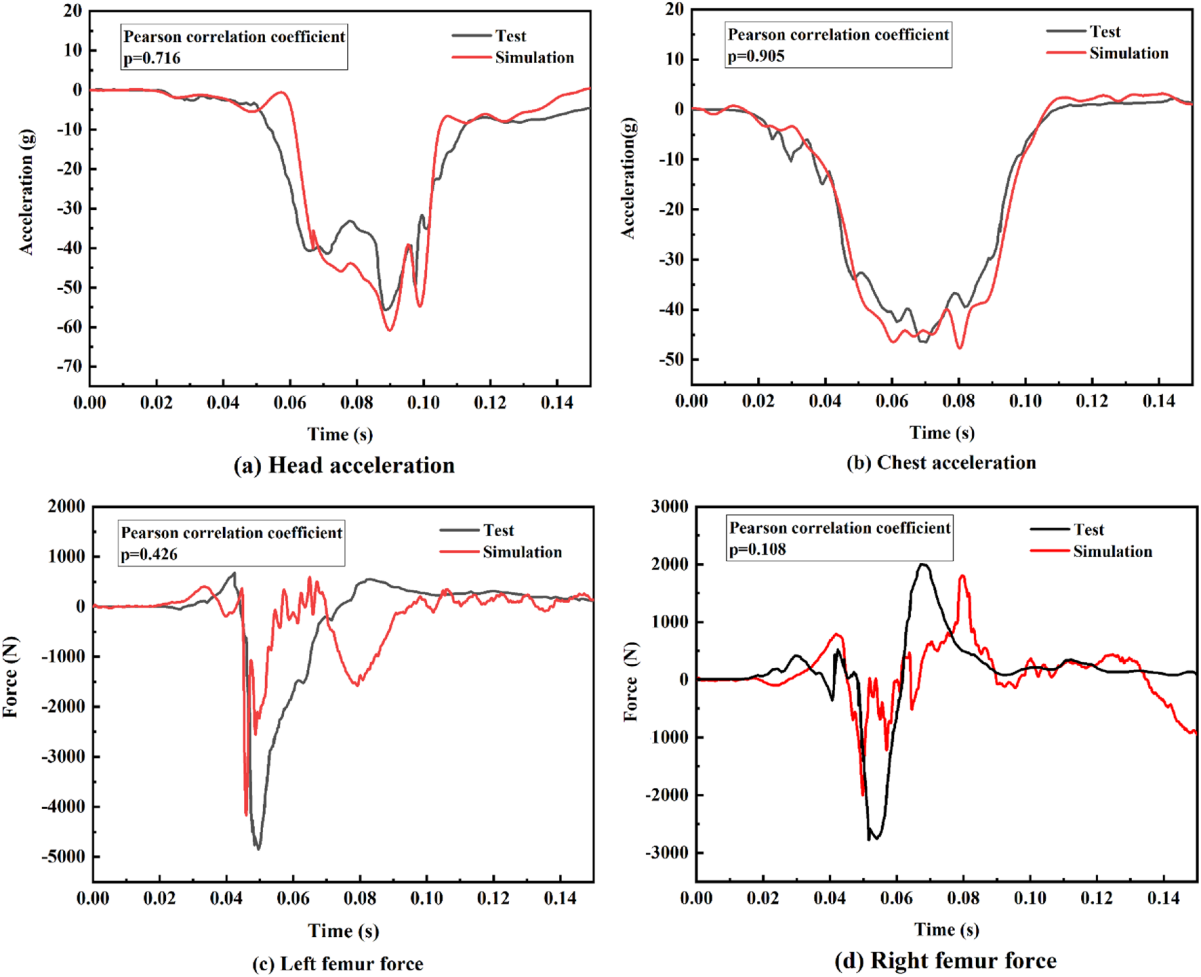
Comparison of experimental and simulated driver injuries.

To verify the restraint system FE model, the B-pillar acceleration obtained from the 35-mph frontal 100% rigid barrier crash simulation was input to the driver-side restraint system model in accordance with the NHTSA NCAP report. The results are presented in Fig. 4; the overall curve trends, driver head, and chest acceleration peaks, and axial forces on the left and right femur are largely consistent with the experimental data from the actual vehicle, demonstrating the validity and stability of the driver-side restraint system model developed in this study. Hence, the model was determined to be valid for subsequent simulation testing.

#### Occupant injury assessment

Due to the complexity of the human body’s structure, the characteristics of impact-related injuries differ between body parts; hence, injury assessment criteria differ between body parts. The Federal Motor Vehicle Safety Standard (FMVSS) 208 regulations stipulate detailed head, neck, chest, and femur injury criteria^33^. The head injury criterion (HIC) of *HIC*_*36*_ with an injury threshold of 1000 is used to evaluate occupant head injury. The Neck Injury Index (*N*_*ij*_) assesses the severity of neck injuries sustained by vehicle occupants. It is determined by measuring the shear force *F*_*X*_, transverse tension *F*_*Y*_*,* and extension moment *M*_*ocy*_, and *N*_*ij*_ = 1 is usually used as the allowable threshold for the neck injury. Chest injuries are evaluated using two metrics: Chest 3 ms acceleration (*C*_*3ms*_) and chest compression (*C*_*comp*_). The acceptable threshold for *C*_*3ms*_ is 60 g, while C_comp_ should be less than 76 mm. The femur is primarily subjected to the impact loading of the dashboard; hence, the axial force on the femur is typically used to assess occupant femur injury, and the FMVSS specified that the femur can tolerate an axial compression force of 10 kN.

In order to obtain a view of the overall occupant injury, the Weighted Injury Criteria (WIC) assessment method is used to assess occupant injuries. It takes into account injuries to all parts of the body, including injuries to the head, chest, and femur. The greater the WIC value, the more severe the occupant's injuries^34,35^. It is calculated as follows:

1 $$WIC=0.6\left(\frac{{HIC}_{36}}{1000}\right)+\frac{0.35\left(\frac{{C}_{3ms}}{60}+\frac{{C}_{comp}}{0.0762}\right)}{2.0}+0.05({F}_{left}^{femur}+{F}_{right}^{femur})/20.0$$

In this formula, ${F}_{left}^{femur}$ and ${F}_{right}^{femur}$ are the left and right femur axial force, respectively, in kilonewtons.

#### Setup for simulation

**Figure 5:**
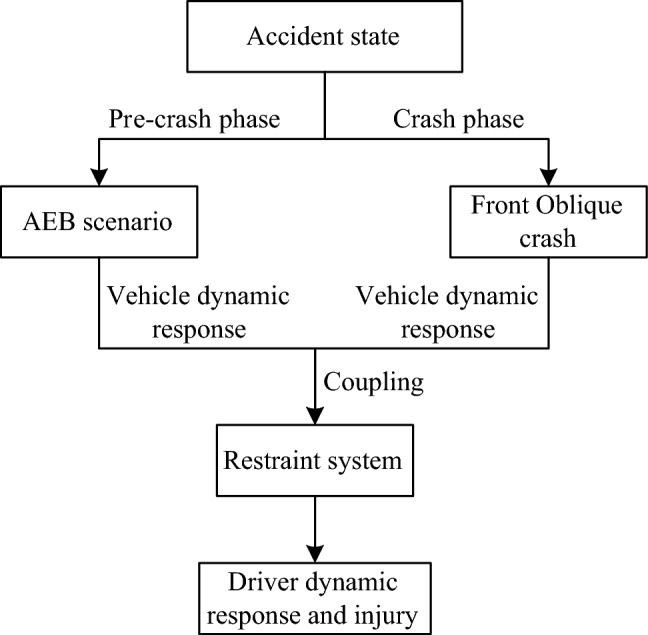
Simulation flowchart.

Figure 5 shows the simulation flowchart. The simulation time is 630 ms, divided into two phases: the first 500 ms is the pre-crash phase, and the following 130 ms is the crash phase. The vehicle dynamic responses of the two phases are studied separately and then combined with the vehicle dynamic responses of the two phases, which are used as the boundary conditions for the inputs of collision conditions. Among them, the AEB scenario was built in Prescan, the frontal oblique collision model and the driver-side restraint system model were built by HyperMesh, and finally, the finite element model was solved by using LS-Dyna to obtain the injury and kinematic response of the occupants.

**Figure 6:**
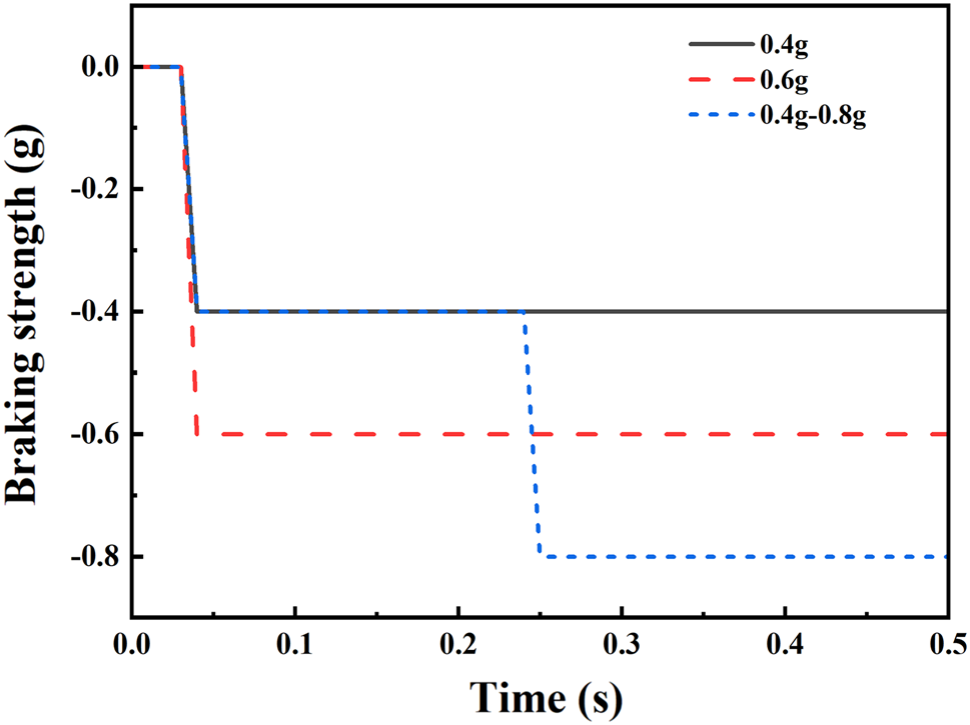
AEB braking strength.

Statistical analysis of traffic accidents has shown that the collision angle of a vehicle forward oblique angle collision is around 30°, so the collision angle set in this model is 30°^36,37^. Based on previous studies, we established three AEB strategies with different deceleration levels (0.6, 0.8, and 0.4-0.8 g) and three collision overlap rates (25%, 50%, and 75%)^38,39^. These AEB strategies are shown in Fig. 6. We simulated nine different collision conditions by combining the AEB strategies with collision overlap rates. Based on this, the effects of pre-tensioning time and pre-tensioning amount of active seat belts on driving injuries were investigated; five pre-tensioning times (0, 100, 200, 300, and 400 ms) and seven pre-tensioning volumes (0, 20, 40, 60, 80, 100, and 120 mm) were set, the baseline pre-tensioning time and pre-tensioning volume were chosen to be 50 ms and 60 mm.

## Results

### Driver kinematic response and injury

#### Effect of AEB on driver kinematic response

**Figure 7:**
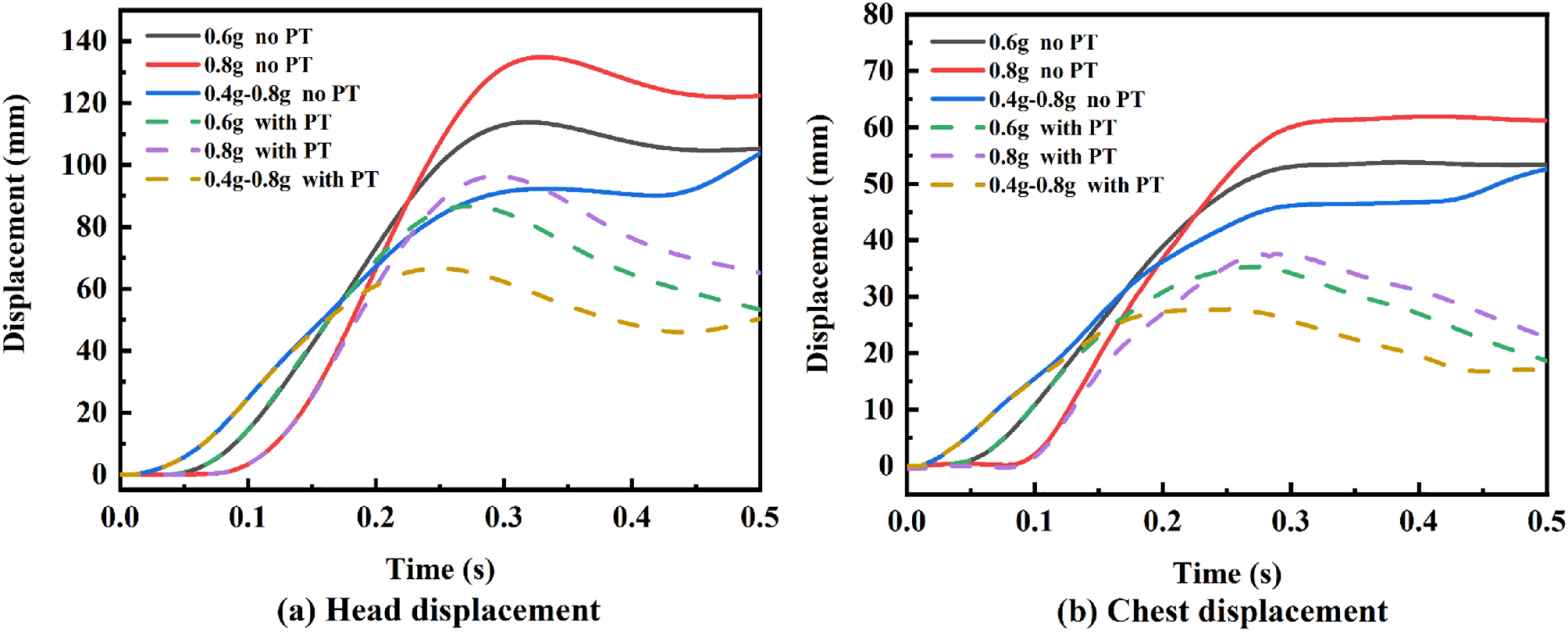
Precrash driver kinematic response. Solid line, no PT; dotted line, with PT.

In the precrash stage, the AEB intervention resulted in an OOP driver. Figure 7 presents the kinematic response of the driver’s head and chest in this phase. As braking caused deceleration over time, the driver’s head and chest displacements increased. Without active seatbelt pre-tensioning, the head and chest forward displacements were considerably higher. Once the active pre-tensioning seat belt was in action, the driver's head displacement was reduced by approximately 49.2%, and the chest displacement was reduced by approximately 66.6%. This shows that the active pre-tensioning seat belts significantly suppressed occupant displacements. Moreover, we found that the driver’s head response lagged their chest response for the pretensioned seatbelt.

#### Driver injury in the integrated simulation

**Figure 8:**
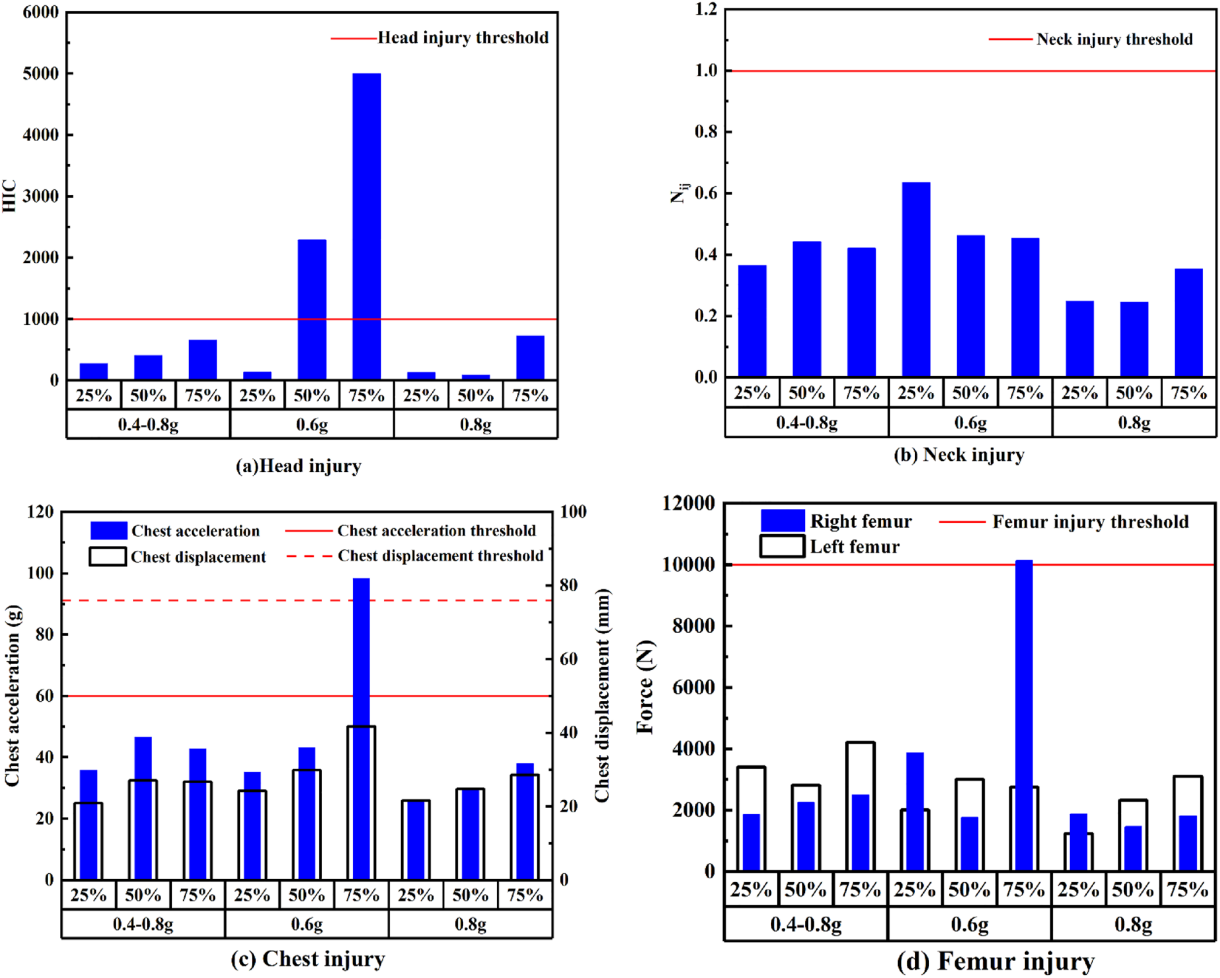
Simulated driver injuries: (**a**) head, (**b**) neck, (**c**) chest, and (**d**) femur.

Each combination of the braking deceleration and overlap velocity factors was simulated, producing nine simulations in total. Driver head, neck, chest, and thigh injuries are presented in Fig. 8. The driver injury trends differ between the various braking deceleration and crash overlap rate combinations. The head injury exceeds the regulatory limit for 0.6 g braking deceleration with 50% and 75% crash overlap, indicating a potentially fatal head injury (Fig. 8a). For neck injury and a given crash overlap, greater braking deceleration is weakly correlated with less severe neck injury; this may be attributable to the neck injury calculation method (Fig. 8b). Figure 8c reveals that at a given overlap rate, more severe injury is likely if braking deceleration is 0.6 g; in particular, this injury may be fatal if the crash overlap rate is 75%. Moreover, at a given braking deceleration, a higher crash overlap rate is related to a more severe chest injury. The results for femur injuries are displayed in Fig. 8d; lower femur force is discovered for a braking deceleration of 0.8 g, and the femur force values are relatively stable for braking decelerations of 0.4-0.8 g. However, femur force is extremely unstable at 0.6 g braking deceleration; hence, leg injuries are difficult to predict. This may be attributable to the lack of driver leg restraints.

#### Effects of an active seatbelt on driver injury

The results presented in the previous section reveal that greater braking deceleration tends to result in less severe driver injury overall; by contrast, increasing the crash overlap rate increases the severity of driver injury. Various methods can be applied to improve braking performance to meet these deceleration requirements, but the vehicle crash overlap rate is difficult to control. Therefore, active seatbelt optimization was performed by assuming an achievable braking deceleration of 0.8 g and a worst-case crash overlap rate of 75%.

#### Effect of pre-tensioning time on driver injury

**Figure 9:**
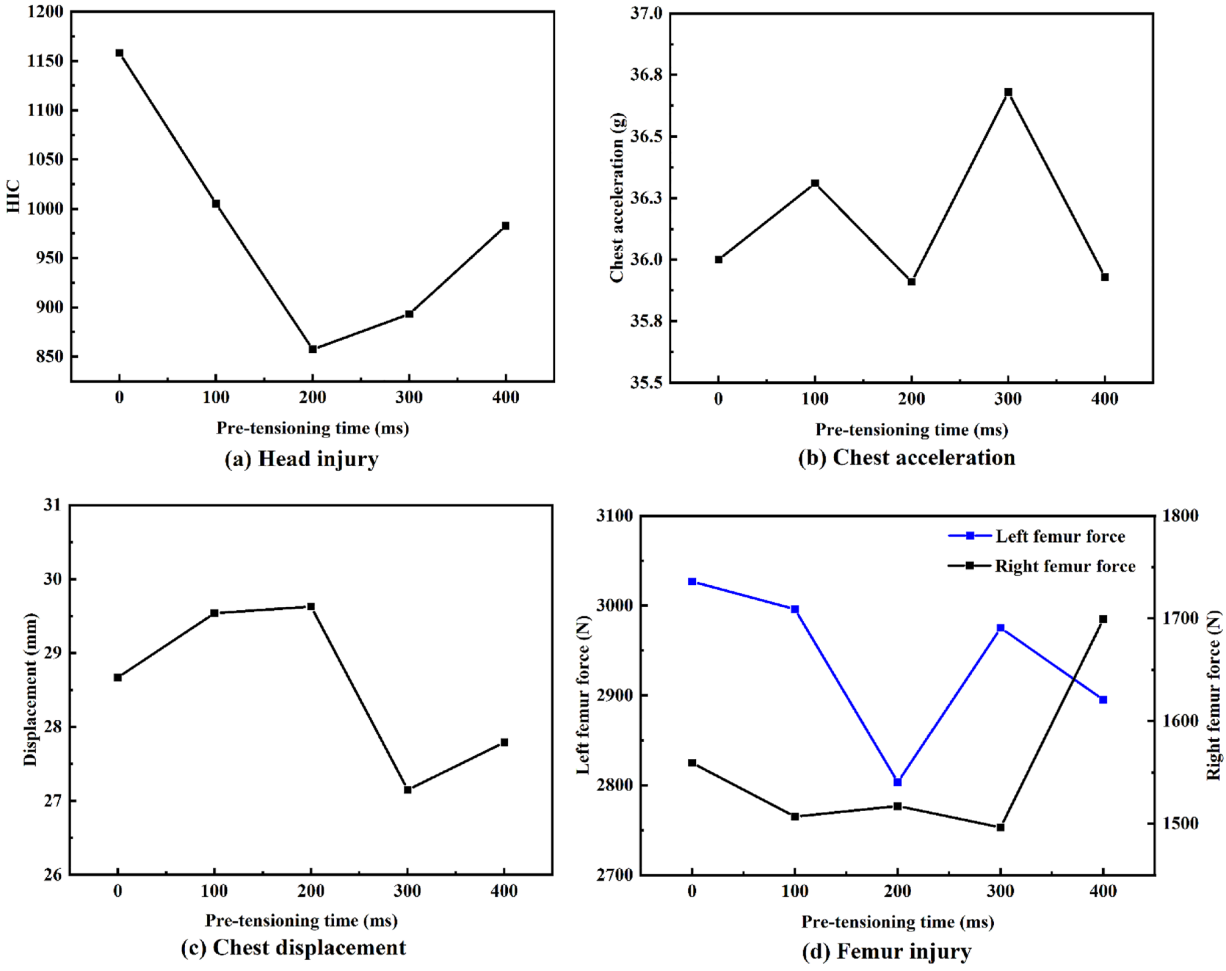
Driver injury for various pre-tensioning times.

The optimal pre-tensioning time was investigated with a constant pre-tensioning amount of 60 mm. Figure 9 reveals that changing the pre-tensioning time affects the severity of injury to all body parts. The HIC and femur force values have clear minima; however, the chest injury trend showed fluctuations within a small range, which may be related to the single force limit we set.

The WIC values for five pre-tensioning times are presented in Table 1. The driver WIC values have a clear minimum at 200 ms; compared to the WIC value obtained by starting the pre-tensioning at 0 ms, a 20.4% reduction was achieved. Larger WIC values were attributed to the driver’s head impacting the steering wheel; head injury has the greatest weight in the WIC value calculation formula, meaning that potential head injuries dominate the results.

**Table 1:** WIC values for various pre-tensioning times.

Pre-tensioning time	0 ms	100 ms	200 ms	300 ms	400 ms
WIC	0.877	0.788	0.698	0.716	0.770

#### Effect of pre-tensioning amount on driver injury

**Figure 10:**
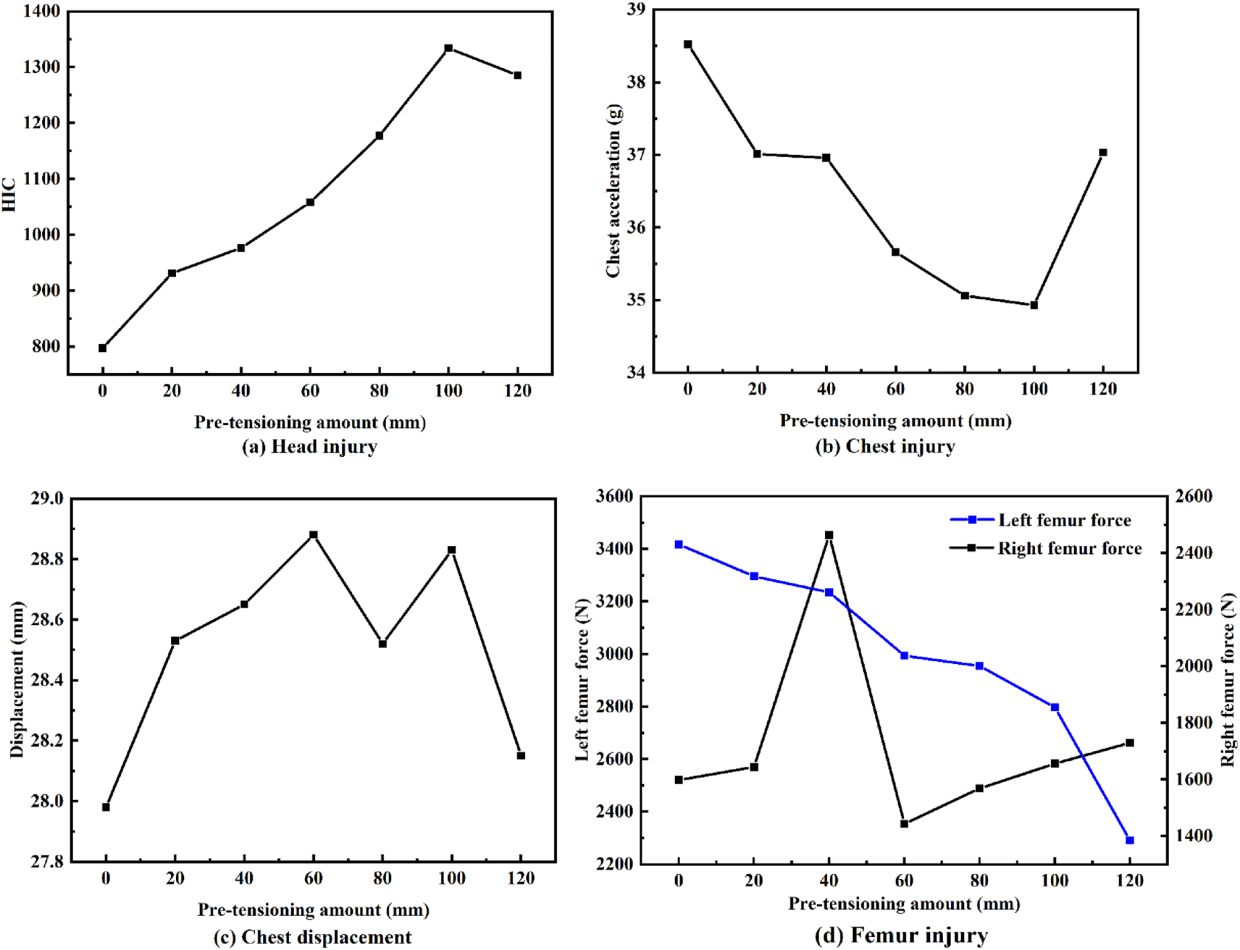
Driver injury for various pre-tensioning amounts.

Various pre-tensioning amounts were investigated under a fixed pre-tensioning time of 50 ms (Fig. 10). The HIC increases as the pre-tensioning amount increases and is highest for a pre-tensioning amount of 100 mm. The chest acceleration decreases as the retracted seatbelt amount increases and is lowest at a pre-tensioning amount of 100 mm; however, chest compression fluctuates within a small range. The left femur force decreases as the pre-tensioning amount increases, but the right femur force is lowest for a pre-tensioning amount of 60 mm. Due to these conflicting results, the WIC was used to identify the parameters resulting in the minimum overall driver injury.

Because head injury has the greatest WIC weight, the WIC and HIC values have similar trends (Table 2). Hence, increasing the seatbelt pre-tensioning amount also increases the WIC. This result was attributed to the airbag ignition time, which was not matched to the seatbelt tensioning, resulting in the driver’s head impacting the steering wheel. In the discussion section, methods of matching an active pre-tensioning seat belt to airbag ignition timing are explored.

**Table 2:** WIC values for various pre-tensioning amounts.

Pre-tensioning amounts	0 mm	20 mm	40 mm	60 mm	80 mm	100 mm	120 mm
WIC	0.667	0.744	0.774	0.816	0.885	0.980	0.954

## Discussion

The integrated simulation indicated that an AEB braking strategy exerting 0.8 g of deceleration effectively minimizes the head and neck injuries of the driver for a specific crash overlap rate. While an AEB braking strategy ranging from 0.4 to 0.8 g substantially reduces driver out-of-position (OOP) incidents, the severity of injuries may still be considerable. This outcome is likely influenced by the initial crash speed. When AEB detects an impending collision and applies 0.8 g braking deceleration, both the initial crash speed and crash energy are reduced, consequently diminishing the severity of the driver's injuries. Therefore, the choice of AEB control strategy is critical to the effectiveness of AEB braking, influencing the potential to either prevent or mitigate a collision.

Future advancements in AEB control strategies should incorporate comprehensive real-world dynamics involving human-vehicle-road-environment interactions to enhance their reliability and safety. In scenarios where a collision is inevitable, the AEB system should aim to decrease crash energy, thereby reducing the extent of damage to both the vehicle and its occupants.

**Figure 11:**
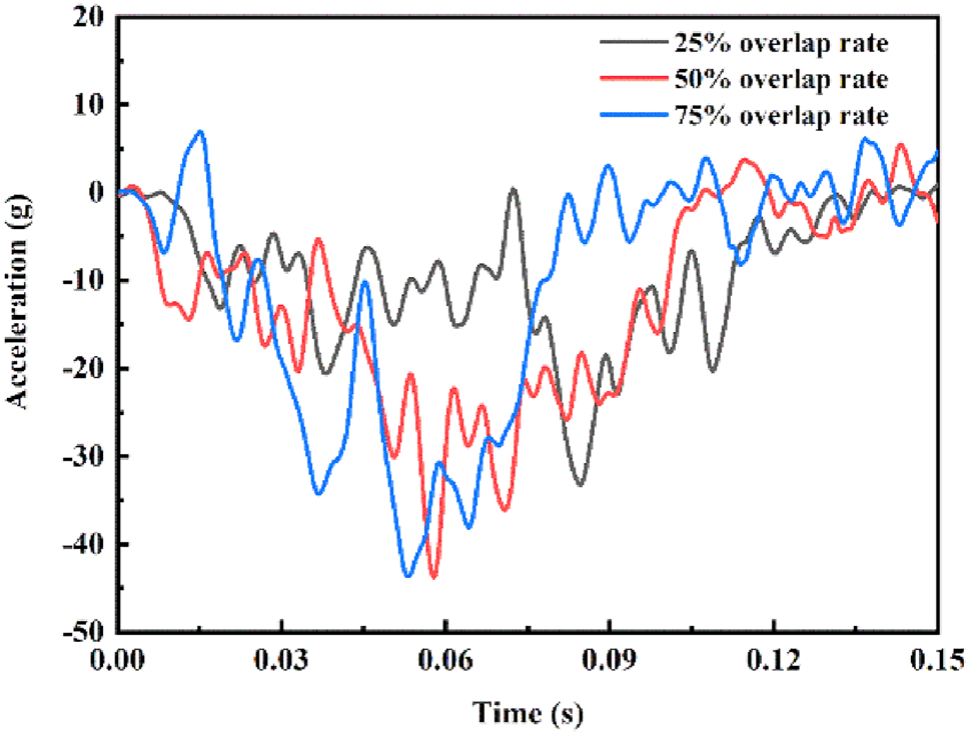
Acceleration for three crash overlap rates.

The results also indicated that driver injuries are severe and even exceed regulatory injury tolerance limits if the crash overlap rate is 75%. The crash acceleration waveform of the vehicle is presented in Fig. 11 for each crash overlap rate. The curve for the crash overlap rate of 75% has two large peaks, which occur early and have low attenuation rates. Current crash regulations only stipulate requirements for crash overlap rates of 25%, 40%, 50%, and 100%^40–42^; hence, the energy-absorbing structure designs of vehicles are optimized only for such crashes and not for a nonstandard 75% crash overlap rate. Hence, vehicles have poor performance in terms of structural crashworthiness and occupant protection at this overlap rate.

**Figure 12:**
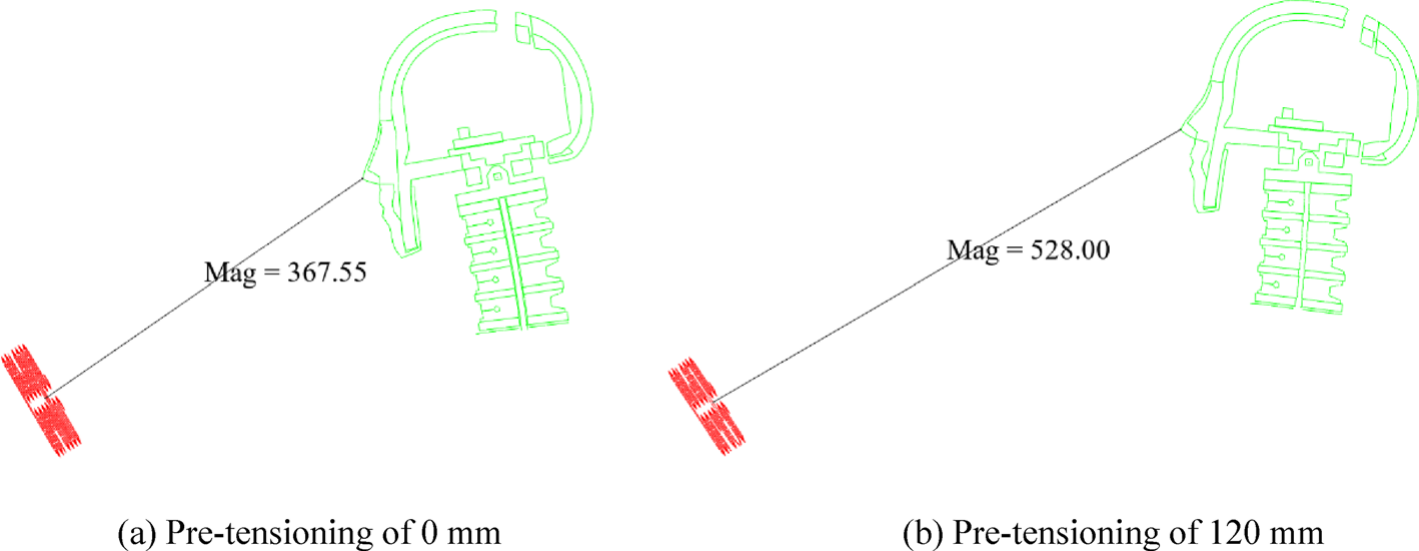
Head-airbag distance for seatbelt pre-tensioning of 0 and 120 mm. Red, airbag; green, driver.

A study of the active seatbelt pre-tensioning amount and driver injury reported the unexpected result that less seatbelt pre-tensioning is correlated with a smaller HIC. Head injury is related to the initial distance between the head and the airbag^43^; this distance is affected by the active seatbelt pre-tensioning amount. Figure 12 presents a comparison of the head-airbag distance after seatbelt pre-tensioning of 0 and 120 mm; these distances were 367.55 mm and 528.00 mm, respectively. Greater seatbelt pre-tensioning results in a larger head-airbag distance.

**Figure 13:**
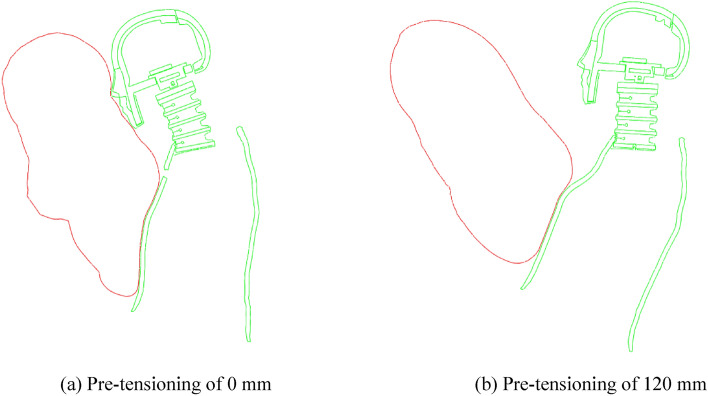
Driver-airbag contact.

Differing initial distances between the head and the airbag led to varied timings of head contact with the airbag. As illustrated in Fig. 13, with the preload set at 0 mm, the driver's head was observed to contact the airbag at *t* = 568 ms. Conversely, when the pre-tensioning amount was adjusted to 120 mm, the driver's head had not yet contacted the airbag at this same timepoint. In the simulation model, both the timing of airbag deployment and the rate of deflation were constant. This implies that a larger initial distance before head-airbag contact results in the airbag having already released most of its gas, thereby diminishing its effectiveness in protecting the driver's head. This phenomenon potentially explains the increase in the HIC value when the pre-tensioning amount is set to 120 mm.

**Figure 14:**
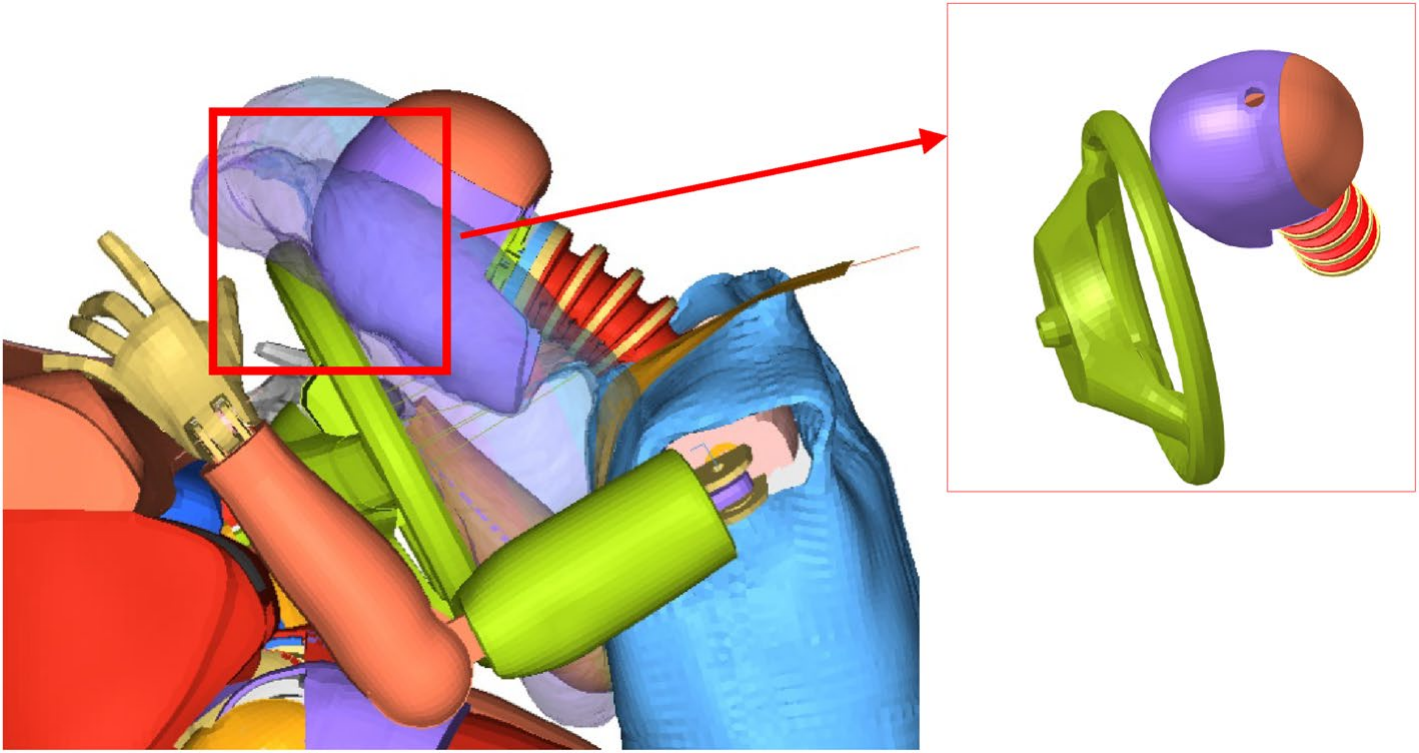
Airbag breakdown. The airbag’s internal pressure is insufficient to support the driver's head, resulting in the head colliding with the steering wheel behind the airbag and drastically increasing the HIC.

Head injury caused by contact with an airbag is affected by several factors^44,45^, such as the airbag’s trigger timing, vent area, airbag volume, and airbag gas mass flow during inflation. Premature airbag triggering results in the airbag stiffness decreasing while the driver is in contact with the airbag, decreasing the support offered by the airbag and resulting in airbag “breakdown” (Fig. 14). However, delayed airbag triggering shortens the distance between the driver and the airbag, resulting in a higher impact force of the airbag on the driver and greater injury severity.

To avoid airbag breakdown, try to delay the airbag trigger time by 10, 20, and 30 ms with a pre-tensioning amount of 120 mm. The driver injuries are presented in Table 3, and the results show that delayed airbag triggering significantly reduced the HIC, with the driver's WIC value being minimized at a 20 ms delay and a 38.7% decrease in WIC value relative to no delay in airbag ignition time. Clearly, delayed airbag triggering time effectively matched active seat belts, reduced the severity of the head injury, and improved safety compared to the original system.

**Table 3:** Comparison of injury to the driver.

Pre-tensioning amount	0 mm	120 mm			
Airbag ignition state	No delay	No delay	Delay 10 ms	Delay 20 ms	Delay 30 ms
HIC	797.06	1288.35	789.96	660.10	877.68
Chest acceleration (g)	38.52	37.05	39.36	40.60	40.12
Chest displacement(mm)	27.98	28.15	27.25	25.50	24.40
Left/right femur force (N)	3417/1598	2268/1731	2804/1934	2851/1768	2920/1954
WIC	0.667	0.954	0.663	0.585	0.712

While the study demonstrated a reduction in driver injury through the adjustment of airbag ignition timing, it also encountered certain limitations. The optimization of active seat belt parameters was conducted without considering the influence of multiple interrelated factors, an oversight that should be addressed in future research. Particularly during the experimental design phase, the interplay among various variables needs to be accounted for. Crucially, all elements of the vehicle's safety system, including the seat belt, airbag, seat, and other components, collectively impact the severity of occupant injuries. Substantial variation in the parameters of one component may lead to a mismatch with other system elements. Therefore, future experimental approaches should focus on developing holistic solutions that encompass multiple components, ensuring that the restraint system remains balanced, adaptable, and effective across various occupant protection scenarios.

## Conclusion

The study assessed the effects of collision overlap rate, AEB brake strength, and active seatbelt pre-tensioning parameters on the kinematic response and injury severity of drivers. Results indicated the highest severity of driver injuries occurred at a collision overlap ratio of 70%. In the pre-crash phase, all AEB control strategies led to a dislocated state of the driver. The use of active seatbelts significantly reduced the driver's head and thorax displacements by 49.2% and 66.6%, respectively. Moreover, during the entire collision event, when the seatbelt pre-tensioning time was set to 200 ms, the lowest weighted injury value for the driver was observed, with a 20.4% reduction compared to the WIC value with pre-tensioning initiated at 0 ms. The study also highlighted that a 20 ms delay in airbag ignition time minimized the driver's WIC, showing a 38.7% decrease compared to scenarios with no delay in airbag ignition. These findings provide essential references for the application of active seat belts in AEB scenarios and support the design of active restraint systems in future advanced assisted driving technologies.

## Data Availability

The datasets used and analyzed during the current study available from the corresponding author on reasonable request.
